# A Rare Case of Rotator Cuff Interposition Causing Humeral Head Subluxation Following Anterior Shoulder Dislocation

**DOI:** 10.7759/cureus.27145

**Published:** 2022-07-22

**Authors:** Owen J Lawrence, Emma Poyser, Hemang Mehta

**Affiliations:** 1 Trauma and Orthopaedics, Nevill Hall Hospital, Abergavenny, GBR

**Keywords:** complication, rotator cuff, open reduction, dislocation, shoulder, soft tissue impingement, shoulder pathology, trauma, shoulder dislocation

## Abstract

This case report aims to highlight that not all shoulder dislocations are simple to treat and that early recognition of complications is key in managing these injuries successfully.

We report the case of a 68-year-old gentleman who presented to Accident and Emergency (A&E) following a fall and sustaining an anterior dislocation of his right shoulder. This was reduced under sedation; however, the patient had an ongoing feeling that his shoulder “was not right.” The subsequent investigation demonstrated persistent anterior subluxation of the humeral head with rotator cuff interposition in the glenohumeral joint. This case appears to be the first of its kind to be reported in which the supraspinatus, subscapularis, and long head of biceps were collectively interposed. This was treated operatively with open reduction and rotator cuff repair, even though the procedure was technically difficult due to tissue fibrosis and the formation of adhesions. The patient progressed well and had a good clinical outcome.

This case highlights that rotator cuff interposition following shoulder dislocation is a rare but debilitating complication and is often neglected in initial care. We must recognise that patients are their own experts, and if they report something is “not right,” further investigation and prompt treatment are required.

## Introduction

The glenohumeral joint is the most mobile joint in the body, offering a very large arc of movement. This movement comes at the expense of stability, and, as such, it is also the most commonly dislocated joint. Over 95% of shoulder dislocations displace anteriorly with the mechanism of injury being forced external rotation in an abducted arm causing the humeral head to lever out of the glenoid [2].

Management of a dislocated shoulder entails closed reduction under sedation using recognised reduction manoeuvers. It is very uncommon for closed reduction to fail, and is usually due to a mechanical block which can be composed of bone, labrum, rotator cuff tendon or the long head of the biceps [3].

Here, we present a very rare case of supraspinatus and subscapularis interposition with a posteriorly dislocated long head of biceps following an anterior shoulder dislocation. This case report aims to highlight that not all shoulder dislocations are simple to treat, and that early recognition of complications is key in managing these injuries successfully.

We report the case of a 68-year-old gentleman who presented to Accident and Emergency (A&E) following a fall and sustaining an anterior dislocation of his right shoulder. This was reduced under sedation; however, the patient had an ongoing feeling that his shoulder “was not right.” Subsequent investigations demonstrated persistent anterior subluxation of the humeral head with rotator cuff interposition in the glenohumeral joint. This case appears to be the first of its kind to be reported in which the supraspinatus, subscapularis, and long head of the biceps were collectively interposed [1]. This was treated operatively with open reduction and rotator cuff repair, although the procedure was technically difficult due to tissue fibrosis and the formation of adhesions.

Although the patient progressed well, this case highlights the fact that not all shoulder dislocations are simply treated, and early recognition and investigation of complications are crucial in ensuring a good clinical outcome.

## Case presentation

A 68-year-old gentleman presented to A&E following a fall off a four-foot ladder sustaining an injury to his right (dominant) shoulder. He was otherwise fit and well and sustained no other injuries in the fall. There was no history of dislocation or pre-existing shoulder pathology. Examination revealed that he was holding his shoulder in an adducted position due to pain, with a very limited range of motion. Upon detailed examination, no neurovascular deficit was found.

**Figure 1:**
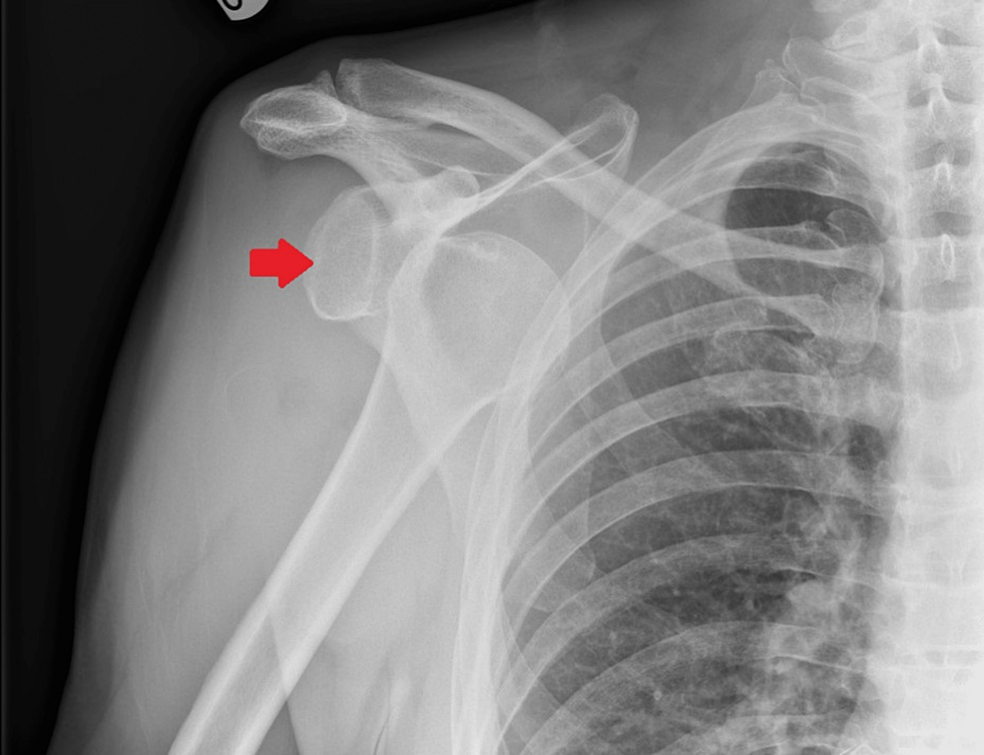
Anteroposterior radiograph of the right shoulder. The arrow indicates anterior dislocation of the humeral head.

**Figure 2:**
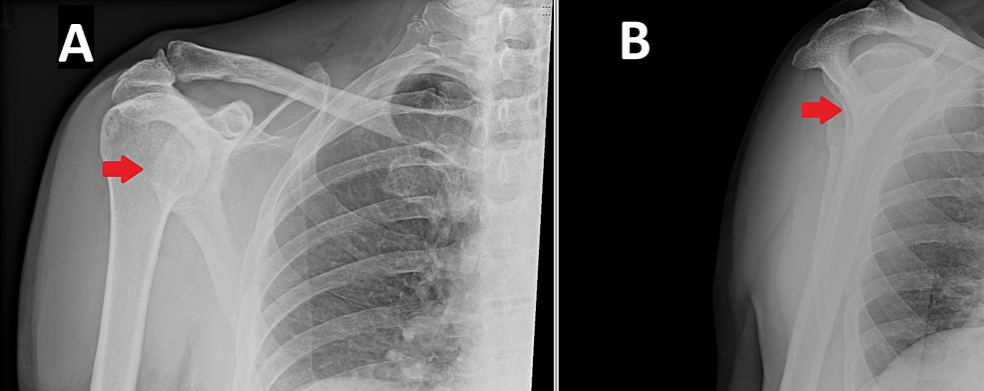
(A) Anteroposterior radiograph of the right shoulder. (B) Lateral Y-view of the right shoulder. The arrows indicate the reduction of the humeral head into the glenoid fossa.

A radiograph, demonstrated in Figure 1, revealed that he sustained an anterior dislocation of his right shoulder with no other obvious bony injury. In A&E, with the use of sedation, the dislocation was reduced using a traction-countertraction technique using a sheet tied around the thorax, positioned at the level of the axilla. The reduction appeared stable and repeat radiographs confirmed relocation, as demonstrated in Figure 2. Despite the reduction, the patient reported that his shoulder “still didn’t feel right.” As per routine practice, he was referred to a fracture clinic.

Investigations

**Figure 3:**
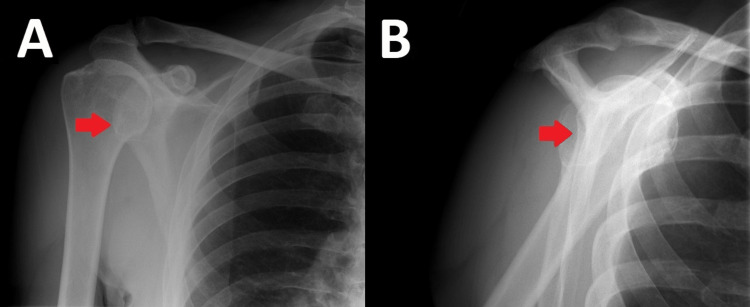
(A) Anteroposterior radiograph of the right shoulder. (B) Lateral Y-view of the right shoulder. The arrows demonstrate anterior subluxation of the humeral head in the glenoid fossa.

The gentleman was reviewed in the fracture clinic nine days later. He reported that his pain had improved; however, his shoulder “did not feel right” to him. Examination revealed a very swollen right shoulder with an abnormal humeral contour. The patient had no active abduction, and passive abduction was extremely painful. A repeat radiograph was taken at this time, displayed in Figure 3, to ensure the humeral head had not re-dislocated. The anteroposterior (AP) radiograph gave the impression that the glenohumeral joint was congruent; however, the Y-view demonstrated that the humeral head was subluxed anteriorly, and this had likely been the case since A&E. A magnetic resonance image (MRI) was arranged with a referral to a specialist orthopaedic shoulder surgeon.

**Figure 4:**
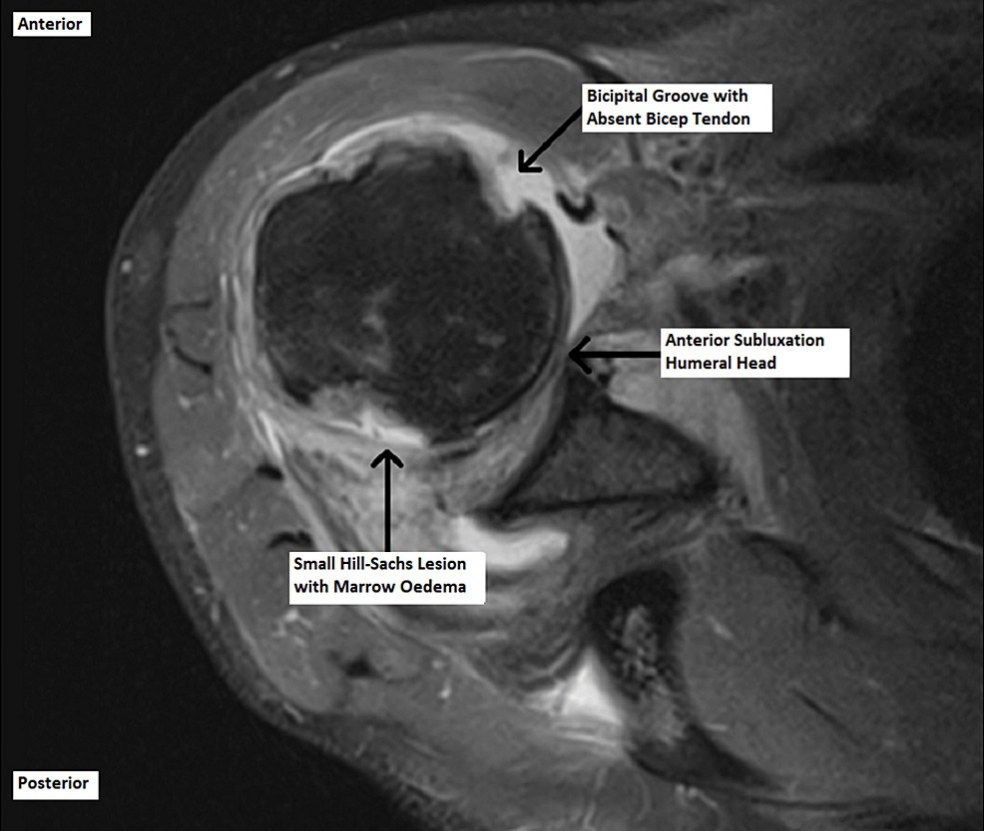
Axial magnetic resonance image of the right shoulder.

**Figure 5:**
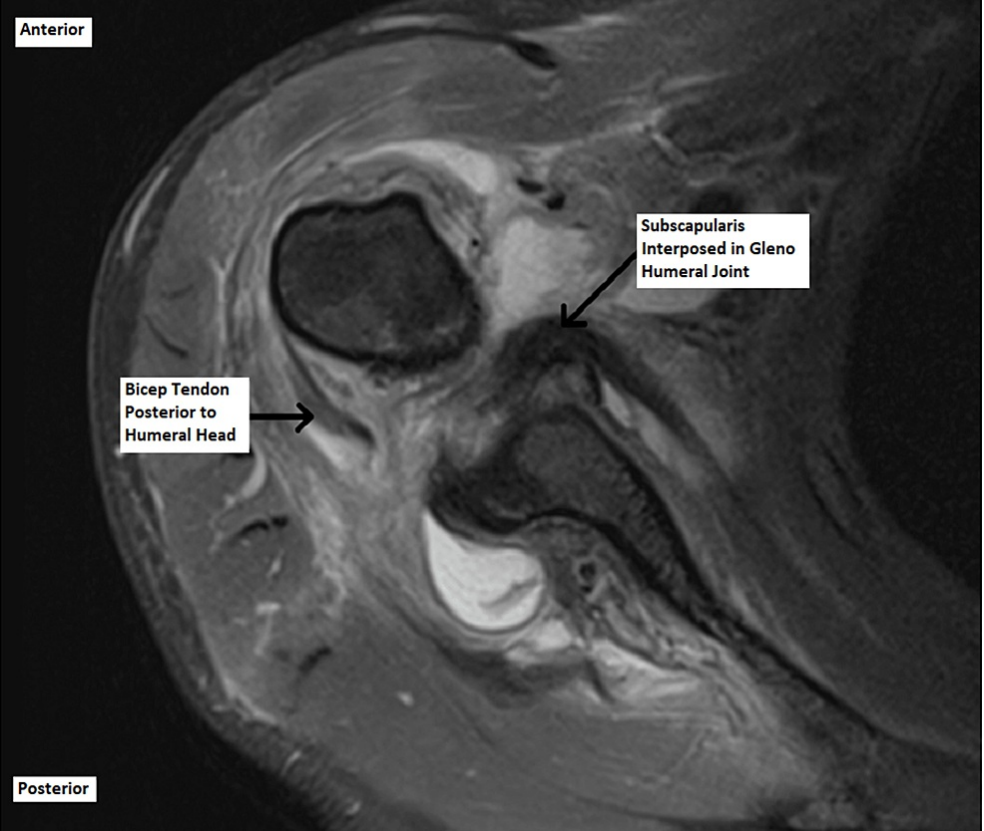
Axial magnetic resonance image of the right shoulder.

**Figure 6:**
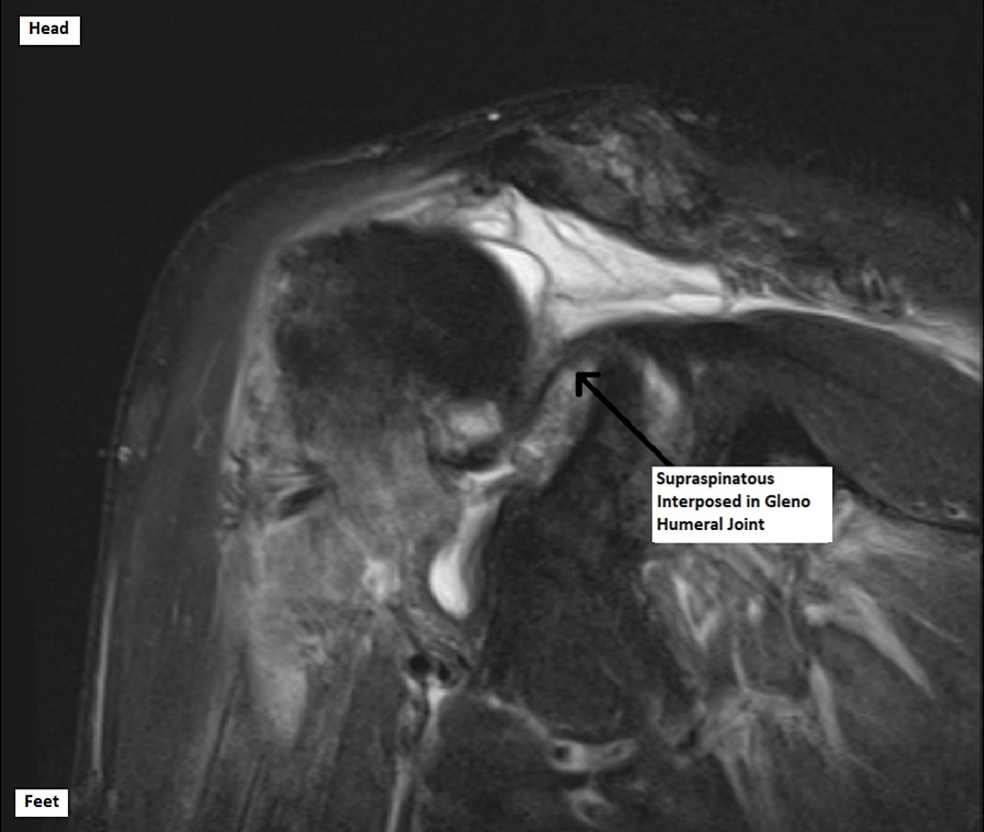
Coronal magnetic resonance image of the right shoulder.

The patient was reviewed in the shoulder clinic six weeks post-injury, and the MRI scan revealed unusual and surprising findings. It demonstrated grossly deranged soft tissues around the right shoulder with an anteriorly subluxed humoral head. There was a full-thickness tear in both supraspinatus and subscapularis tendons, and these were interposed in the glenohumeral joint. In addition, the biceps tendon was dislocated from the bicipital groove and was posterior to the humeral head. These findings are demonstrated in Figures 4-6 below.

Treatment

Following an in-depth discussion with the patient, the decision was made to undergo open reduction, exploration, and repair of the rotator cuff in the right shoulder. The procedure was undertaken eight weeks post-injury.

**Figure 7:**
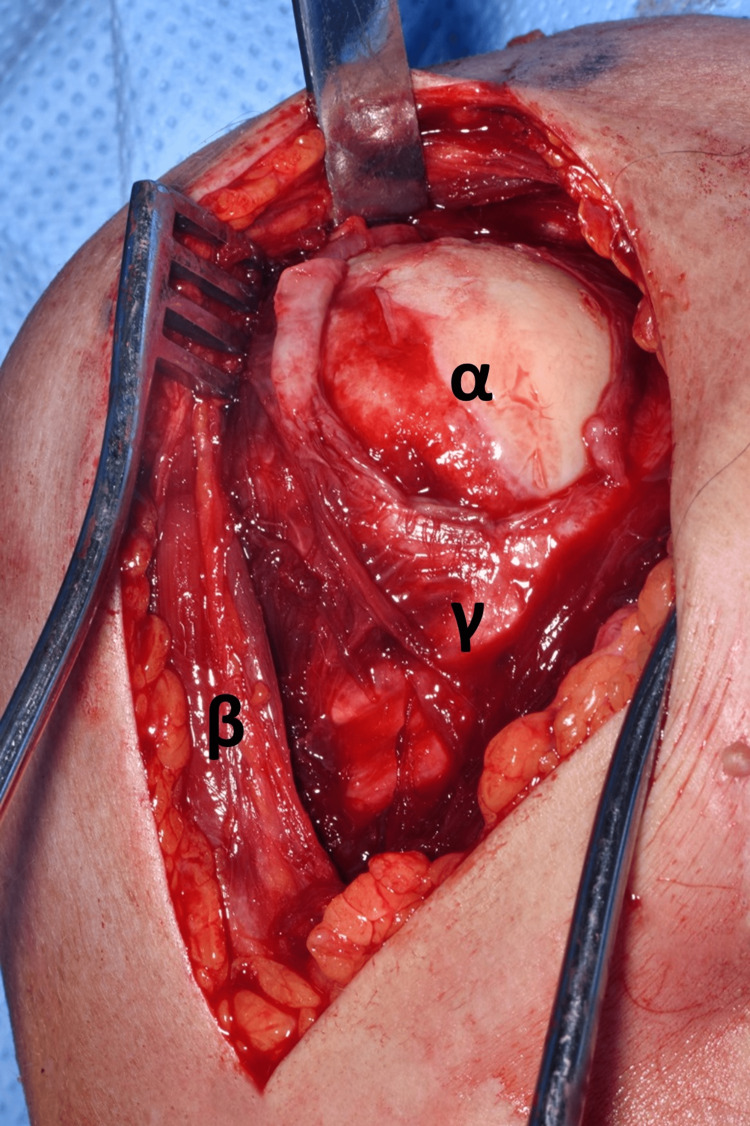
Image demonstrating the humeral head button-holed through the subscapularis muscle. (α) Humeral head. (β) Deltoid muscle. (γ) Subscapularis muscle.

**Figure 8:**
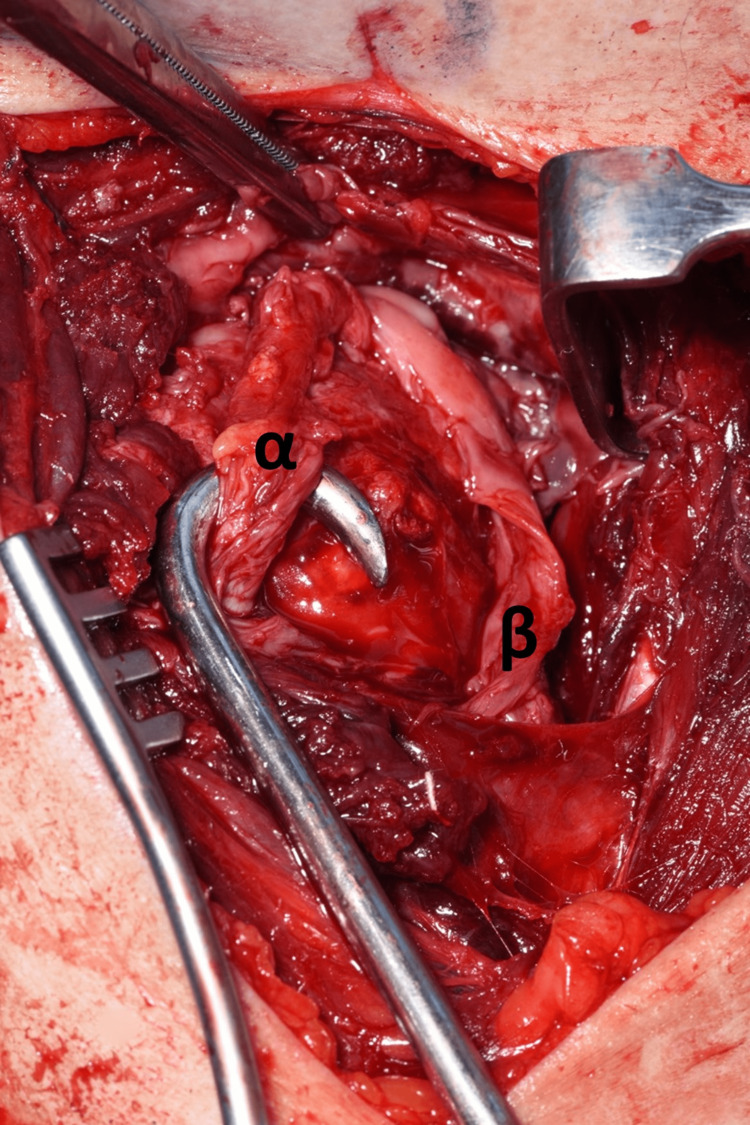
Displaced anatomy identification. (α) Bicep tendon. (β) Subscapularis tendon.

**Figure 9:**
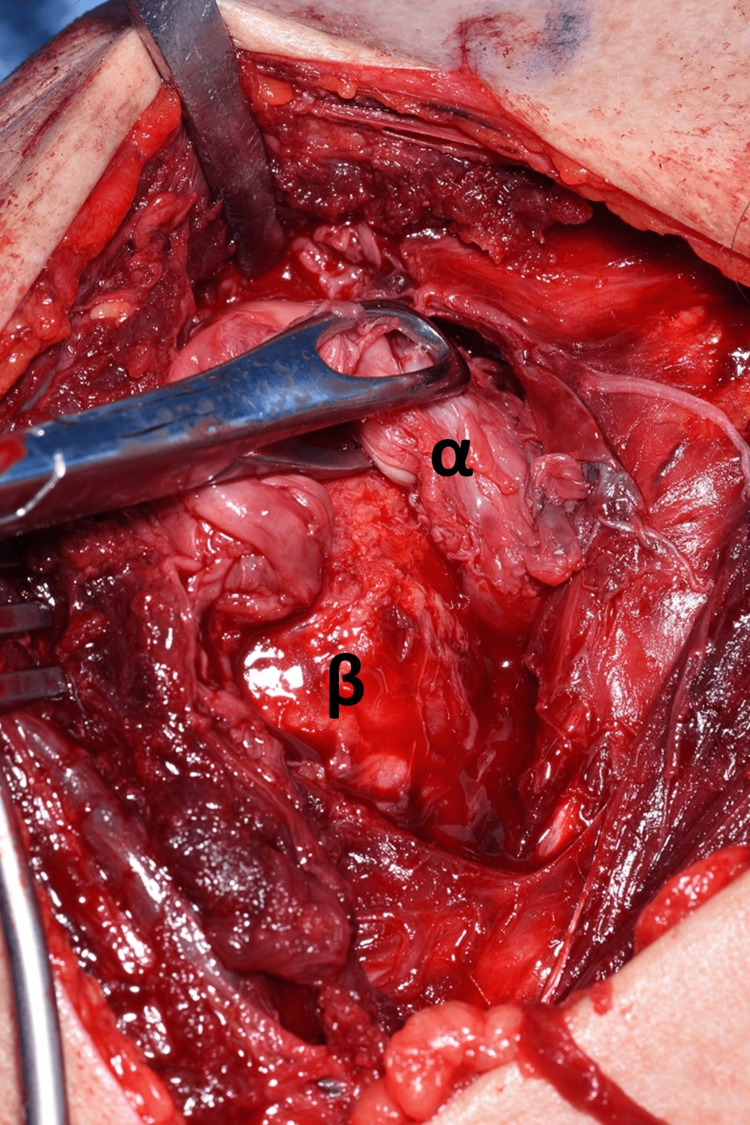
Displaced anatomy identification. (α) Supraspinatus tendon. (β) Humeral head.

The shoulder was approached via the deltopectoral approach, and the humeral head was immediately visible as it had button-holed through subscapularis, as demonstrated in Figure 7. Following this, subscapularis was elevated to approach the humerus, and careful dissection was carried out to locate the bicep tendon posteriorly and the supraspinatus tendon from within the joint. This was very difficult due to fibrosis and formation of adhesions as this was eight weeks post-injury. Figure 8 and Figure 9 demonstrate the identification of these key structures and, subsequently, reduction of the humeral head was possible.

**Figure 10:**
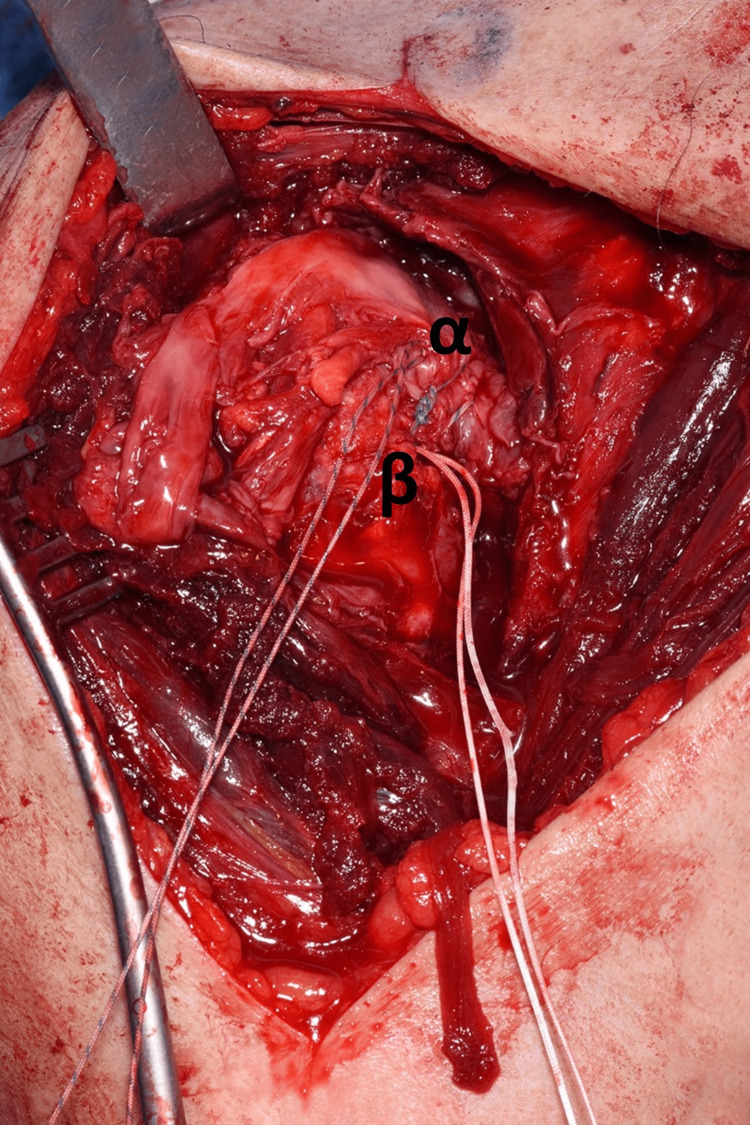
Supraspinatus muscle repair with the use of a bone anchor. (α) Supraspinatus muscle. (β) Bone anchor fixation of the supraspinatus.

Once all the pertinent anatomy was adequately dissected and released, the biceps tendon was relocated into the bicipital groove and two bone anchors were used to fix both the supraspinatus/infraspinatus complex and subscapularis to the proximal humerus securely. Part of this process is demonstrated in Figure 10. Finally, subscapularis was repaired onto the lesser tuberosity ensuring good tissue tension and the wound was closed.

Outcome

**Figure 11:**
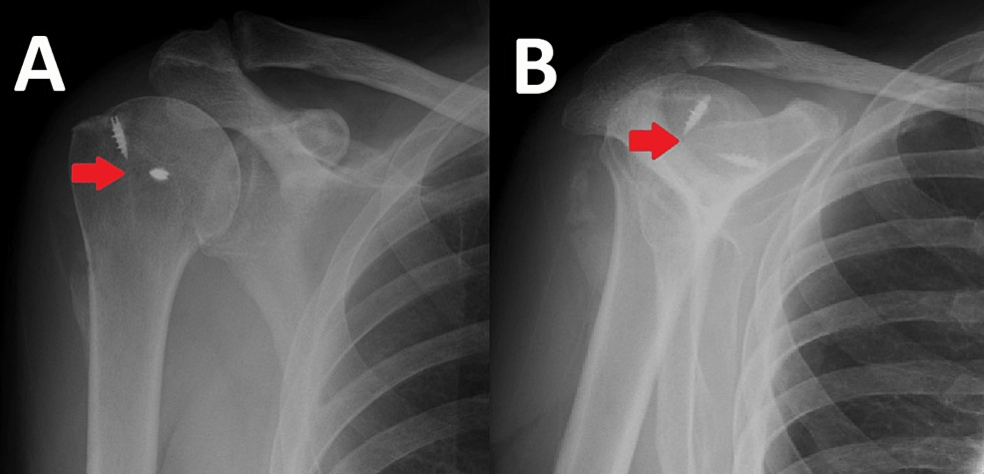
Postoperative radiographs. Arrows indicate in-situ bone anchors in the humeral head. (A) Anteroposterior radiograph of the right shoulder. (B) Lateral Y-view of the right shoulder.

The patient was discharged following a one-night stay and was immobilised in a sling for four weeks with subsequent physiotherapy. His rehabilitation included early passive pendulum exercises with the introduction of limited active movement from week four. The patient was reviewed at eight weeks postoperatively and was progressing very well with his shoulder. His postoperative radiograph, displayed in Figure 11, showed the congruent glenohumeral joint with bone anchors in place. He has already achieved greater than 100 degrees of motion in both forward flexion and abduction and is continuing with physiotherapy. Follow-up is ongoing and we are hopeful that his active range of motion will continue to improve.

## Discussion

Shoulder dislocations are the most common large joint to present to A&E Departments. In the United Kingdom, the incidence of shoulder dislocations is 40/100,000 person-years in males and 14/100,000 person-years in females, and it occurs in a bimodal age distribution [4]. The majority of dislocations are managed via closed reduction with the use of analgesics or sedation.

It is rare for closed reduction to fail, and this is usually due to a bony or soft tissue block. Rotator cuff tears can be present in up to 31% of anterior shoulder dislocations [5]. It is more common in the elderly population with an incidence of up to 80% in those over 60 years old. Persistent subluxation of the humeral head due to the interposition of the rotator cuff is a rare complication, with limited case reports being available in the literature.

Pantazis et al. (2017) performed a literature review of irreducible anterior shoulder dislocations and found 22 reports [1]. The most common block to reduction was the interposition of the long head of the bicep tendon alone or in combination with a greater tuberosity fracture. The second most common cause was avulsion or tear of the subscapularis with interposition. In no case was it reported that the bicep tendon, subscapularis, and supraspinatus were collectively interposed, and, as such, this may truly be the first time an injury of this kind is presented.

In cases of irreducible dislocation, preoperative imaging via MRI/computerized tomography (CT) is essential. There is no gold standard in imaging modality due to the spectrum of causes that may cause the reduction block. CT is indeed excellent at identifying underlying greater/lesser tuberosity fractures, Hill-Sachs lesions, and bony Bankart lesions. Meanwhile, MRI provides detailed knowledge of the surrounding tissue including the rotator cuff tendons and the long head of the bicep tendon. Bencardino et al. (2013) concluded that the decision to use CT, CT arthrography, MR, or MR arthrography depends on each clinical scenario and is based on the history, examination, and X-ray findings [6].

Time to the theatre from injury was eight weeks in this case, and the procedure was technically demanding. Due to fibrosis and adhesion formation, it was difficult to clearly identify anatomical structures around the shoulder joint, as well as to reduce the humeral head into the glenoid fossa. The British Elbow and Shoulder Society (BESS) Patient Care Pathway (2015) recommends that all unreduced dislocations should undergo urgent reduction, and where suspicion of a rotator cuff tear exists, an urgent referral should be made to the next available shoulder clinic appointment [7].

As this is an unusual case, no specific outcome data are available for long-term function. The American Academy of Orthopaedic Surgeons (AAOS) guidelines on the management of rotator cuff repairs report that evidence is inconclusive as to whether arthroscopic, open or mini-open procedure provides the best outcome [8]. Walton et al. (2012) demonstrated that though arthroscopic techniques may lead to earlier functional outcomes and pain improvement, at two years postoperatively, both groups were comparable [9].

Regarding open cuff repair, Millet et al. (2011) documented repair survivorship of 94% at five years and 83% at 10 years, with a patient satisfaction rating of 8 out of 10 [10]. Shin et al. (2012) similarly reported a case series of 17 patients over 60 years old who required operative intervention for primary shoulder dislocation and reported an average shoulder Constant Score of 78 at the final follow-up [11,12]. Currently, our patient is progressing well with high satisfaction, and it will be interesting to see both his repair survivorship and long-term clinical outcomes.

Through this case, we hope to have raised awareness of the rare but possible complications following shoulder dislocation. Through our experience, we have offered a perspective on the investigation and intervention of rotator cuff interposition. Our key message is to ensure reduction of dislocation is confirmed in two planes radiographically, and in case of any concern, early investigation and referral to a shoulder specialist will lead to the best clinical outcome.

## Conclusions

Through this case, we hope to have raised awareness of the rare but debilitating complication of rotator cuff interposition following shoulder dislocation. It can often be neglected in initial care, and, as such, we need to remember that not all dislocations are simple injuries and a high index of suspicion is always needed. We must ensure that following any joint relocation, the reduction is confirmed in two planes to ensure joint congruency. In our case, the patient was aware something was “not right,” and this underlines the principle that patients are their own experts and further investigation was warranted. Through our experience, we have offered a perspective on the investigation and operative intervention of rotator cuff interposition. Our key message is to definitively ensure reduction of dislocation is confirmed radiographically, and in case of any concern, early investigation and referral to a shoulder specialist are best. This pathway will ensure adequate management and the best possible patient outcome.
